# The relationship between role stress, psychological empowerment and organizational silence of psychiatric nurses: a variable-centered and individual-centered analysis

**DOI:** 10.3389/fpsyt.2026.1916837

**Published:** 2026-09-30

**Authors:** Yangguang Li, Shumin Gao, Lina Xie

**Affiliations:** Nursing Department, Shenyang Mental Health Center, Shenyang, China

**Keywords:** organizational silence, potential profile analysis, psychiatric nurses, psychological empowerment, role stress

## Abstract

**Background:**

In psychiatric nursing, organizational silence behavior seriously affects patient safety and nursing quality. Role stress is an important predictor of organizational silence, and psychological empowerment can alleviate role stress and reduce silence behavior. However, the mediating relationship among these three variables and group heterogeneity remain unclear.

**Objective:**

To explore the mediating role of psychological empowerment between role stress and organizational silence, and to identify different latent classes of the “role stress–psychological empowerment” dyad using latent profile analysis, so as to provide evidence for targeted interventions.

**Methods:**

In the Mental Health Center of Shenyang City, Liaoning Province in the northeastern part of China, 391 nurses were included in a cross-sectional survey. Subsequently, the mediating effect was first tested using the structural equation model, and then potential subgroups were identified using latent profile analysis.

**Results:**

(1) Role stress was positively correlated with organizational silence, while psychological empowerment was negatively correlated with both role stress and organizational silence. (2) Role stress not only directly affected organizational silence but also had an indirect effect through psychological empowerment, with psychological empowerment playing a partial mediating role and the mediation effect accounting for 23.6% of the total effect. (3) The role stress–psychological empowerment dyad was classified into three latent classes, and organizational silence scores differed significantly across classes.

**Conclusions:**

Psychological empowerment serves as a partial mediator; the “role stress–psychological empowerment” dyad demonstrates distinct classification characteristics. It is recommended that nursing managers adopt differentiated intervention strategies according to group characteristics to reduce organizational silence and foster a participative and positive organizational climate.

## Introduction

The current healthcare environment is becoming increasingly complex, with frequent medical disputes and patient safety incidents (1). Healthcare institutions urgently need employees to actively voice their suggestions to optimize processes and reduce the occurrence of adverse events (2). Nurses, as the largest group in the healthcare system and those with the closest contact with patients, are often the first to detect potential risks in clinical practice and propose improvements based on their practical experience (3). However, a growing number of studies have shown that the phenomenon of “silence” is common in the nursing profession (4, 5). That is, nurses choose to withhold their opinions due to psychological or practical factors and do not speak up actively, a phenomenon termed nurses’ organizational silence (6). According to a survey, 91.2% of nurses have experienced organizational silence (7). This behavior not only directly affects work engagement, reduces job satisfaction and work performance, but also leads to interruption of information transmission at the organizational level, increasing the probability of unsafe events (8, 9).

Current research on nurses’ organizational silence behavior has mainly focused on general hospitals (4, 10), with insufficient attention to the specialized field of mental health. The clinical context of psychiatric nurses is rather unique, as their daily work often involves a high risk of violence (11). Studies have shown that frequent workplace violence can directly induce organizational silence behavior among nurses (12). Moreover, in closed psychiatric wards without family accompaniment, patient safety is the top priority (13), and organizational silence itself has been confirmed as a factor that seriously threatens patient safety (14). Therefore, in psychiatric nursing practice, organizational silence behavior among specialist nurses may be more prominent and deserves attention and exploration.

Multiple studies have indicated that role stress is a key predictor of organizational silence; when employees experience higher levels of role stress, they are more likely to exhibit organizational silence behavior (15, 16). Role stress refers to the psychological stress individuals feel when they have difficulty meeting role expectations due to role conflict, ambiguity, or overload (17). Psychiatric nurses undertake multiple roles in their work, such as treatment implementer, safety manager, emotional supporter, and external liaison (18, 19), which increases their psychological burden. In addition, the high level of emotional labor and occupational stigma in psychiatry can lead to increased negative emotions among nurses, further exacerbating psychological stress (20, 21). Under prolonged role stress, nurses may adopt “silence” as a coping strategy to avoid conflict or reduce further stress. Role theory suggests that the roles individuals occupy in the social structure influence their behaviors, attitudes, and self-perceptions (22). This theory is mainly applied in organizational behavior to analyze the impact of role expectations in the workplace on employees’ work behaviors (23). Therefore, based on this theory, this study proposed the first hypothesis (H1): role stress is positively correlated with organizational silence behavior in psychiatric nurses.

Psychological empowerment refers to a composite of psychological states and cognitions experienced by individuals, often manifested in organizations as individuals’ intrinsic motivation and perceived sense of control over their work role (24). Previous studies have shown that psychological empowerment is an important psychological resource. Employees with a high level of psychological empowerment can not only alleviate the impact of role stress (25) but also reduce organizational silence behavior (26). However, whether low levels of psychological empowerment mediate the relationship between role stress and organizational silence has not yet been explored. This study used the Job Demands-Resources (JD-R) model as a theoretical framework to deeply examine the relationships among the three variables. According to the JD-R model (27), an individual’s work status depends on the dynamic balance between job demands and available resources. Job resources can buffer and mitigate the impact of job demands, ultimately guiding employees toward positive work behavior patterns. Conversely, an increase in job demands or a lack of job resources leads to negative work outcomes. Therefore, the model proposes a dual-path hypothesis: when negative work outcomes occur, two coping pathways can be taken—one is to reduce job demands, and the other is to increase job resources (28). Role stress, as a type of job demand, may gradually deplete nurses’ level of psychological empowerment, inhibit their motivation to express opinions, and thus induce organizational silence behavior. Accordingly, this study proposed the second hypothesis (H2): role stress is negatively correlated with psychological empowerment, and psychological empowerment is negatively correlated with organizational silence behavior, serving as a mediator in psychiatric nurses.

Previous studies have mostly adopted a variable-centered perspective to examine average relationships among variables, which to some extent assumes that the research population is homogeneous (29). However, role stress and psychological empowerment are both subjective inner perceptions of individuals, and different combinations of these experiences may exhibit qualitative differences. Such within-group heterogeneity is crucial for understanding the complexity of phenomena and for formulating precise management measures (30); moreover, the latent profile analysis method is characterized by rigor, accuracy, and objectivity in its criteria for retaining the number of classes (31). This study will additionally incorporate a person-centered perspective and employ latent profile analysis to identify naturally occurring subgroups based on different combination patterns of role stress and psychological empowerment, and to examine differences in the levels of organizational silence behavior across these classes. This approach can transcend the understanding of the “average sample,” truly delve into the interior of the group, and reveal vulnerable high-risk populations for silence. Therefore, this study proposed the third hypothesis (H3): there are distinct latent classes of “role stress-psychological empowerment,” and individuals in different latent classes exhibit differences in their levels of organizational silence.

In summary, first, under the joint guidance of role theory and the JD-R model, this study integrates the “role–behavior” and “demands–resources” frameworks into the high-risk psychiatric nursing context, to examine whether psychological empowerment, as a critical job resource, mediates the relationship between role stress and organizational silence. Second, by combining variable-centered and person-centered perspectives, and building on the validated mediating effect, this study further reveals the heterogeneous differentiation among different latent classes of nurses, thereby providing a targeted empirical basis for clinical management interventions.

## Materials and methods

### Participants

This study adopted a cross-sectional design. From January 2025 to June 2025, the researchers selected the largest psychiatric hospital in Northeast China, located in Shenyang, Liaoning Province, and used cluster sampling to recruit all psychiatric nurses in this hospital as the study population. Inclusion criteria were: (1) holding a valid nurse practice certificate; (2) working in clinical frontline psychiatric care for at least one year. Exclusion criteria were: (1) nurses who were not working in the hospital during the survey period due to leave or off-site training; and (2) intern nurses or nurses undergoing training.

### Sample size

First, according to Kendall’s sample size estimation criterion (32), the sample size can be 5 to 10 times the number of variables. This study involved 19 variables, and a 20% invalid response rate was considered; therefore, the minimum required sample size ranged from 114 to 228. Second, Schumacker et al. (33) suggested that a sample size between 200 and 500 is needed to obtain more stable models when constructing structural equation models, with larger samples leading to greater model stability. Finally, referring to relevant literature, the minimum sample size for latent profile analysis studies is 300 (34). The number of participants surveyed in this study met all the above criteria.

### Data collection

After obtaining approval from the hospital administrators and the nursing department, the researchers adopted a top-down approach. Ultimately, head nurses from different departments distributed the electronic questionnaires in their respective departmental work group chats. A standardized instruction was provided at the beginning of the questionnaire, stating the inclusion and exclusion criteria, explaining the purpose and significance of the study, and assuring confidentiality and anonymity. Participants could start answering after providing informed consent. After the survey, two researchers cross-checked the data. A total of 436 nurses were targeted. Based on the inclusion and exclusion criteria, 418 questionnaires were returned. After excluding invalid questionnaires in which responses to all items were identical, answers showed obvious regular patterns, or the response time was less than three minutes, a total of 391 valid questionnaires were finally obtained, yielding an effective response rate of 93.54%.

### Ethical statement

Before the study was initiated, ethical approval was obtained from the Ethics Committee of Shenyang Mental Health Center Hospital (approval No. 2024006). The study also followed the Strengthening the Reporting of Observational Studies in Epidemiology (STROBE) statement.

### Instruments

#### Demographic information

Based on previous literature (35, 36), the researchers designed a general information questionnaire, which included the following items: gender, age (years), marital status, professional title, educational level, years of working in psychiatric nursing, whether holding a managerial position, and employment type.

#### Role stressors scale

The original scale was developed by Rizzo et al. in 1970, and subsequently revised by Peterson et al., forming the Role Stressors Scale (RSS). It was later translated and adapted by Chinese scholars Li Chaoping et al. (37). The scale consists of three dimensions and 13 items: role ambiguity (5 items), role conflict (3 items), and role overload (5 items). A 5-point Likert scale was used, with scores from 1 to 5 representing “strongly disagree” to “strongly agree”. The total score ranges from 13 to 65, with higher scores indicating higher levels of role stress. The scale has been validated among Chinese nurses (38). In this study, the Cronbach’s α coefficients for each dimension ranged from 0.79 to 0.9, all above 0.7, indicating good internal consistency. Furthermore, factor analysis showed that the factor loadings of all 13 items exceeded 0.4, confirming the robust construct validity of the scale (39).

#### Psychological Empowerment Scale

This study used the Chinese version of the Psychological Empowerment Scale developed by Spreitzer et al. and translated/revised by Li Chaoping et al. (40). The scale consists of four dimensions and 12 items: meaning (3 items), competence (3 items), self-determination (3 items), and impact (3 items). A 5-point Likert scale was used, with scores from 1 to 5 representing “strongly disagree” to “strongly agree”. The total score ranges from 12 to 60, with higher scores indicating higher levels of psychological empowerment among psychiatric nurses. This scale has been applied to psychiatric nurses in China and has shown good reliability and validity (41). In this study, the Cronbach’s α coefficient for the total scale was 0.84, and the Cronbach’s α values for the subscales ranged from 0.73 to 0.85. The high factor loadings of the items on their respective dimensions (> 0.4) further confirm the scale’s robust construct validity (40).

#### Nurses’ organizational silence behavior questionnaire

This questionnaire was developed by Yang Jing et al. (42) in 2016 and consists of four dimensions and 20 items: acquiescent silence (6 items), defensive silence (6 items), prosocial silence (4 items), and indifferent silence (4 items). A 5-point Likert scale was used, with scores from 1 to 5 representing “never feel” to “always feel”. The total score ranges from 20 to 100, with higher scores indicating higher levels of nurses’ silence. Several scholars have applied it to the Chinese nurse population with good applicability and reliability (43). In this study, the Cronbach’s α coefficient for the total scale was 0.912, and the Cronbach’s α coefficients for the subscales ranged from 0.829 to 0.933. All items demonstrated high factor loadings (> 0.4) on their respective dimensions, supporting the scale’s robust construct validity (4).

### Statistical analysis

First, the data were imported into Excel for organization. Then, SPSS 27.0 was used for statistical analysis, including Harman’s single-factor test, descriptive analysis, and Pearson correlation analysis. On this basis, Amos 29.0 was used to construct a structural equation model, and the Bootstrap method was employed to test the mediation effect. Finally, during the potential profile analysis (LPA), the items of the three sub-dimensions of the role stress scale were evenly distributed, and the items of the four sub-dimensions of the psychological empowerment scale were also evenly distributed. A total of 7 continuous variables were input into Mplus 8.3 for analysis as the observation indicators. This was done to explore the differences in organizational silence behaviors of mental health nurses under different “role stress - psychological empowerment” categories. All statistical tests were considered statistically significant when *P* < 0.05.

## Results

### Common method bias test

Data were collected using the self-report method, and the Harman single-factor test was used to assess the confounding bias. The results showed that the eigenvalues of all 14 factors were greater than 1. The first factor explained 32.26% of the variance, which was below the 40% threshold (44). Therefore, the confounding bias was considered negligible, and the research results were deemed robust.

### General demographic characteristics

The basic characteristics of the psychiatric nurses in this study are shown in **Table 1**. The mean age was(36.5 ± 9.23) years. Among the sample, 67% were female nurses and 33% were male nurses; 86% of the nurses held a bachelor’s degree; 74% were married. The most common range of psychiatric nursing experience was 5~9 years, accounting for 38% of the participants. Regarding professional titles, there were 76 nurses (primary level), 133 junior nurses, 144 supervisor nurses, and 38 nurses with senior titles or above. Nurses holding a position of team leader, head nurse, or above accounted for 29% of the sample. Nurses with permanent employment status accounted for 40%.

**Table 1:** Demographic characteristics of psychiatric specialist nurses (n=391).

Demographic variables	Category	Frequency	Percentage(%)
Gender	Male	129	32.99
	Female	262	67.01
Age	≤25	28	7.16
	26%35	159	40.66
	36%45	139	35.55
	≥46	65	16.62
Education	Junior college degree	50	12.79
	undergraduate degree	336	85.93
	Master’s degree or above	5	1.29
Marital status	Unmarried	82	20.97
	Married	290	74.17
	Others	19	4.86
Professional title	Nurse	76	19.44
	Nurse practitioner	133	34.02
	Senior Nurse Practitioner	144	36.83
	Deputy chief nurse and above	38	9.72
Years of work experience in a psychiatric hospital	<5	45	11.51
	5%9	148	37.85
	10%19	126	32.23
	>20	72	18.41
Having or not having a position	No	277	70.84
	Nursing team leader	75	19.18
	Head Nurse	39	9.97
Employment method	Compilation	158	40.41
	Contracts and others	233	59.59

### Mean scores and correlation analysis of role stress, psychological empowerment, and organizational silence behavior among psychiatric nurses

The results showed that the mean score of role stress among psychiatric nurses was (40.84 ± 5.25), the mean score of psychological empowerment was (41.79 ± 5.75), and the mean score of organizational silence behavior was (47.89 ± 9.99). Detailed information is presented in **Table 2**. The Pearson correlation analysis results showed that role stress was positively correlated with organizational silence behavior (*r* = 0.657, *P* < 0.01) and negatively correlated with psychological empowerment (*r* = -0.417, *P* < 0.01); psychological empowerment was negatively correlated with organizational silence behavior (*r* = -0.621, *P* < 0.01). More information is shown in **Table 2**.

**Table 2:** Descriptive analysis and correlation analysis.

Variables	Mean ± standard deviation	RS	PE	OS
RS	40.84 ± 5.25	-		
PE	41.79 ± 5.75	-0.417**	-	
OS	47.89 ± 9.99	0.657**	-0.621**	-

**indicates *P*<0.01. RS, Role Stress; PE, Psychological empowerment; OS, Organizational silence.

### Relationship between role stress, psychological empowerment, and organizational silence behavior among psychiatric nurses: a variable-centered analysis

Based on the theoretical hypothesis model, role stress was set as the independent variable, psychological empowerment as the mediator, and organizational silence behavior as the dependent variable. A structural equation model was constructed using Amos 29.0 for the three study variables (see **Figure 1**). The analysis results showed that the model had good fit indices, and the contents of the report are all standardized numerical values: *χ*²=84.197, *df* = 41, *χ²/df* = 2.054, CFI = 0.990, GFI = 0.962, AGFI = 0.938, TLI = 0.986, NFI = 0.980, IFI = 0.990, RMSEA = 0.052, SRMR = 0.036. The path analysis results showed that role stress had a significant negative effect on psychological empowerment (*β* = -0.352, *P* < 0.001), psychological empowerment had a significant negative effect on organizational silence behavior (*β* = -0.312, *P* < 0.001), and role stress had a significant positive direct effect on organizational silence behavior (*β* = 0.354, *P* < 0.001). The detailed path diagram is presented in **Table 3**.

**Figure 1:**
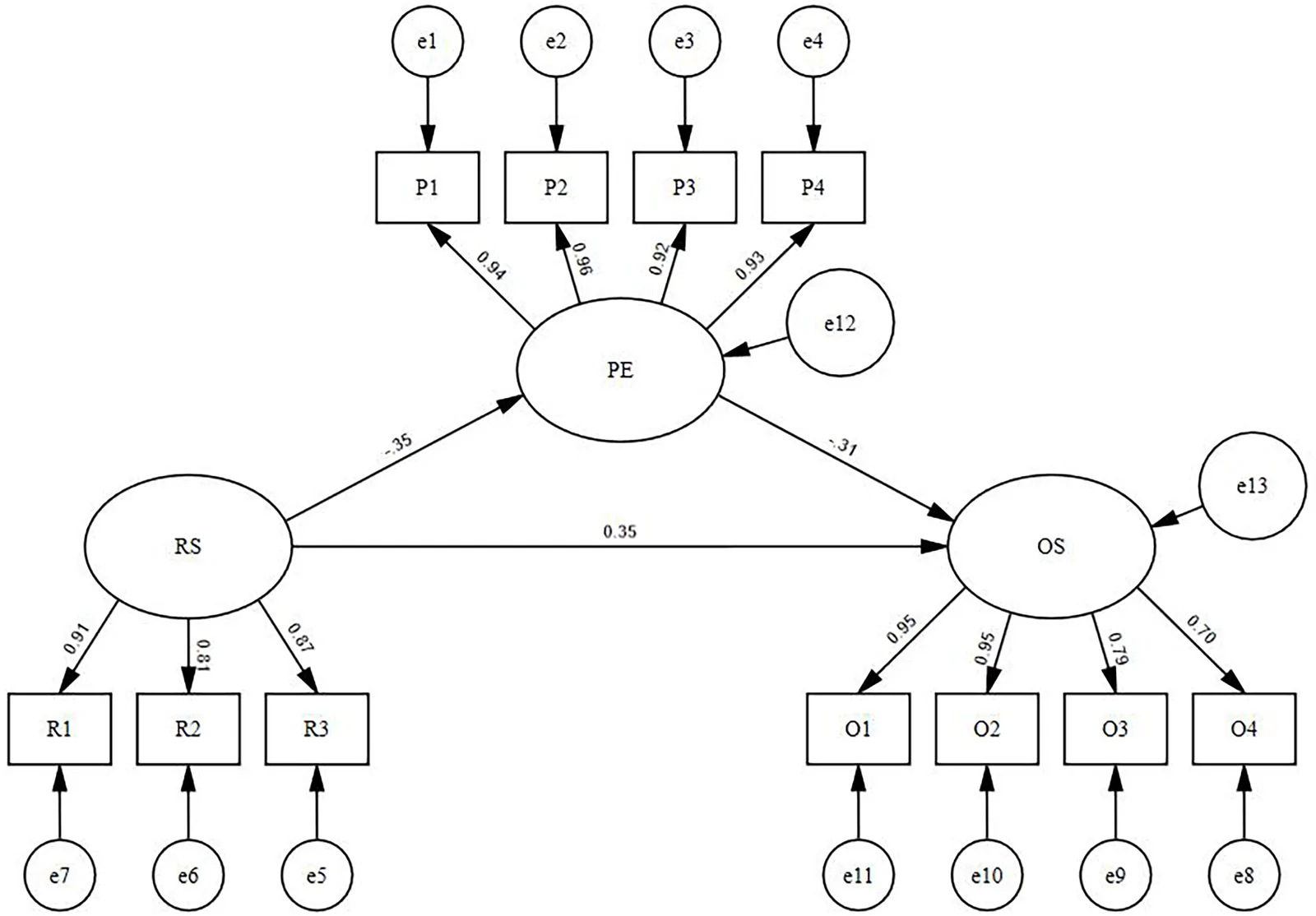
Path diagram of structural equation model. RS, Role Stress; PE, Psychological empowerment; OS, Organizational silence. (Standardization coefficient).

**Table 3:** Path analysis results.

Path	Standardized coefficient	SE	CR	*P*
Role stress→Psychological Empowerment	-0.352	0.039	-6.740	***
Psychological Empowerment→Organizational silence	-0.312	0.092	-6.393	***
Role stress→Organizational silence	0.354	0.073	6.919	***

***indicates *P*<0.001.

To rigorously test the mediating effect, a bias-corrected non-parametric percentile Bootstrap sampling method (Bias-corrected percentile bootstrap method) was further employed. The sampling was repeated 5000 times and the 95% confidence interval (CI) was calculated. If the CI does not include 0, it is considered that the effect is significant. As shown in **Table 4**, the indirect effect value of psychological empowerment between role stress and organizational silence was 0.110, with the 95% CI excluding 0; the direct effect value of role stress on organizational silence behavior was 0.354, with the 95% CI excluding 0; and the total effect value was 0.464. These results indicate that psychological empowerment plays a partial mediating role between role stress and organizational silence behavior.

**Table 4:** Test of the mediating effect of psychological empowerment between role stress and organizational silence.

Item	Route	Effect	Effect quantity	95%CI	
				Lower	Upper
Direct effect	Role stress→Organizational silence	0.354	76.4%	0.261	0.439
Indirect effect	Role stress→Psychological Empowerment→Organizational silence	0.110	23.6%	0.071	0.150
Total effect	–	0.464	100%	0.374	0.531

### Prediction of organizational silence behavior among psychiatric nurses by latent classes of “role stress–psychological empowerment”: a person-centered analysis

Based on seven observation indicators, the three sub-dimensions of role stress (role conflict, role ambiguity, role overload) were correlated with psychological empowerment (job meaning, competence, autonomy, influence). In this study, latent profile analysis was employed to fit models with 1 to 4 latent categories. The fit indices for each model are shown in **Table 5**. Lower AIC, BIC, and aBIC values indicate better model fit. An Entropy value greater than 0.8 indicates high classification accuracy, with values closer to 1 indicating clearer classification. For LMR and BLRT, a *P* value less than 0.05 indicates that the k-class model is superior to the (k-1)-class model (45). As shown in **Table 5**, as the number of classes increased, AIC, BIC, and aBIC gradually decreased. The Entropy values for all models were above 0.8, indicating good classification accuracy. Regarding model selection, both LMR and BLRT were significant for the three-class model (*P* < 0.05), whereas LMR was not significant for the four-class model (*P* = 0.216), suggesting that adding a fourth class did not significantly improve model fit. Considering model parsimony, statistical indices, and theoretical interpretability, the three-class model was determined as the optimal solution (46).

**Table 5:** Summary table of fitting information for potential profile analysis.

Category	AIC	BIC	aBIC	Entropy	LMR(*P*)	BLPT(*P*)	Group size	Class probabilities
1	18095.86	18222.86	18121.32	-	-	-	391	-
2	15520.09	15714.56	15559.08	0.970	<0.001	<0.001	136/255	035/0.65
3	14651.45	14913.39	14703.97	0.969	<0.01	<0.001	59/245/87	0.15/0.63/0.22
4	13858.42	14187.82	13924.47	0.947	0.216	<0.001	72/141/99/79	0.19/0.36/0.25/0.20

The average membership probabilities for each latent class are shown in **Table 6**. The within-class membership probabilities for C1, C2, and C3 were 0.984, 0.988, and 0.979, respectively, all above 0.95, indicating high internal consistency and reliability of the classification results. The score trends of role stress and psychological empowerment across the three latent classes are shown in **Figure 2**. Class 1 (C1) had the lowest role stress scores and the highest psychological empowerment scores (n = 59, 15.09%), and was named the “low stress–high empowerment” group. Class 2 (C2) had moderate scores on both variables (n = 245, 62.66%), and was named the “moderate stress–moderate empowerment” group. Class 3 (C3) had the highest role stress scores and the lowest psychological empowerment scores (n = 87, 22.25%), and was named the “high stress–low empowerment” group. Thus, the three groups showed distinct patterns of combining role stress and psychological empowerment.

**Table 6:** The average attribution probability of each potential participant.

Category	Number of people	Percentage	Posterior probabilities		
			C1	C2	C3
C1	59	15.09%	0.984	0.000	0.015
C2	245	62.66%	0.000	0.988	0.012
C3	87	22.25%	0.003	0.017	0.979

**Figure 2:**
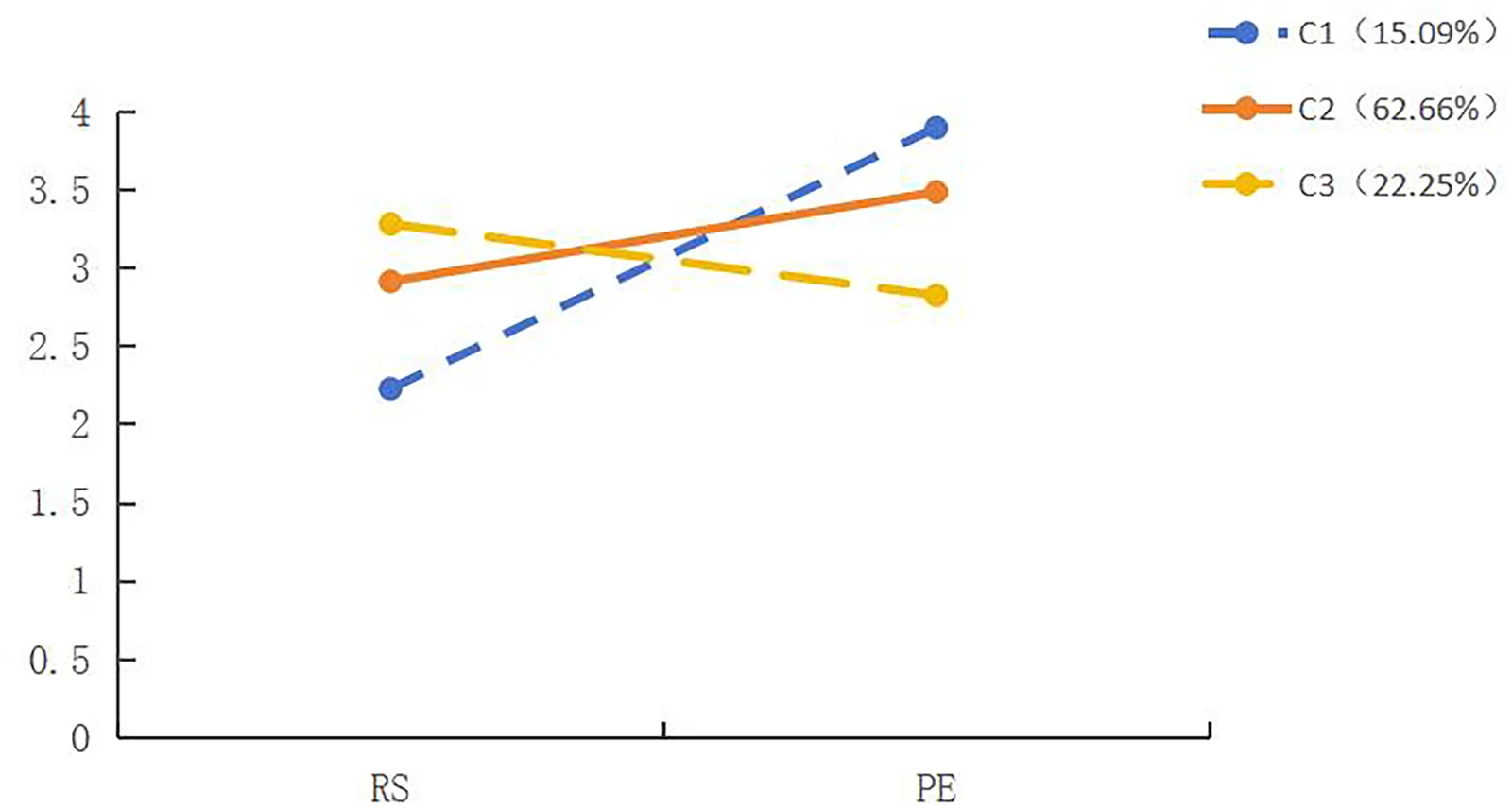
For the X- axis: RS, Role Stress; PE, Psychological Empowerment, Y-axis represents the mean scores of items.

To further investigate differences in organizational silence across the different latent classes, a one-way ANOVA was conducted, with latent class as the independent variable and the total organizational silence score and each dimension as dependent variables. As shown in **Table 7**, significant differences were found among the latent classes in the total organizational silence score (F = 52.371, *P* < 0.001) and in all dimensions (all *P* < 0.001). *Post-hoc* comparisons (Tukey HSD) indicated that the total organizational silence behavior score and the scores for each dimension, from high to low, were as follows: high stress–low empowerment group (C3) > medium stress–medium empowerment group (C2) > low stress–high empowerment group (C1), with all between-group differences reaching statistical significance.

**Table 7:** Comparison of the scores of different potential category role stress - psychological empowerment on each dimension of organizational silence.

	Latent profile(s)			ANOVA	*Post hoc* test
Item	C1	C2	C3		
tacit silence	11.42 ± 2.85	13.71 ± 3.12	16.26 ± 4.13	32.456^***^	C3>C2>C1
defensive silence	13.16 ± 3.36	15.56 ± 4.52	17.31 ± 4.89	46.809^***^	C3>C2>C1
prosocial silence	9.31 ± 2.68	11.53 ± 3.04	14.35 ± 3.62	48.650^***^	C3>C2>C1
indifferent silence	7.54 ± 1.79	9.36 ± 2.07	10.53 ± 2.85	36.447^***^	C3>C2>C1
Total score of organizational silence	40.46 ± 5.36	47.94 ± 7.29	58.32 ± 11.24	52.371^***^	C3>C2>C1

***indicates *P*<0.001.

## Discussion

### Relationship between role stress and organizational silence behavior among psychiatric nurses: the mediating role of psychological empowerment – a variable-centered approach

The findings indicate a significant positive correlation between role stress and organizational silence behavior, validating Hypothesis 1. This finding is consistent with previous research in general workplace settings (15, 16) and provides a new perspective within the specific high-risk context of psychiatric care. A survey covering 17 countries pointed out that nurses in different clinical settings experience different levels of role stress (47). The uniqueness of psychiatric nursing lies in the fact that the patients often have impaired cognition and lack insight, resulting in generally low cooperation with nursing care. During acute episodes of illness, patients may be unable to care for themselves, and in severe cases, may verbally abuse nurses or even exhibit violent behavior (11). Therefore, when experiencing high levels of role stress, although some individuals may adopt active coping strategies, more nurses may choose to remain silent – i.e., exhibit organizational silence – to avoid further conflict, reduce additional workload, or protect themselves. Managers of psychiatric institutions could focus interventions on reducing role stress itself, by establishing clear role boundaries, explicit work procedures, and defined responsibilities, thereby reducing organizational silence.

The results of the mediation analysis showed that role stress not only directly affected organizational silence behavior but also had an indirect effect through the partial mediating role of psychological empowerment, with the mediation effect accounting for 23.65% of the total effect. In addition, the correlation results showed that psychological empowerment was negatively correlated with both role stress and organizational silence behavior, indicating that role stress can indirectly contribute to organizational silence behavior by weakening nurses’ psychological empowerment, thus supporting Hypothesis 2. In this study, the overall psychological empowerment score of psychiatric nurses was lower than that of clinical nurses in general hospitals (48), which is generally consistent with the level reported in previous studies on psychiatric nurses (41). The main reasons may include the long disease course and uncertain prognosis of psychiatric patients, which can lead to a sense of professional powerlessness among nurses; the potential risk of violence, which may limit their autonomy in clinical decision-making; the predominantly custodial and medication-focused nature of work, with relatively few technical nursing procedures, reducing some nurses’ sense of meaning; and social stigma toward mental health, which may further undermine their sense of professional meaning (49, 50). These environmental characteristics contribute to a relatively low level of psychological empowerment among psychiatric nurses, forming a pattern of “high stress – low empowerment – silence”. This finding is consistent with the Job Demands-Resources model, under which demands and resources in the work environment directly influence individuals’ work motivation and behavior (28). As a core personal resource, psychological empowerment is weakened by role stress, making individuals feel a lack of impact, autonomy, and efficacy. Consequently, their motivation to make suggestions or challenge the status quo decreases, and they are more likely to adopt organizational silence behavior to preserve the remaining psychological resources. In the future, nursing managers should recognize the importance of psychological empowerment, grant more responsibilities and authority to enhance psychological empowerment among psychiatric nurses, thereby enabling them to exercise greater autonomy and collaboration, and stimulating their inner strengths to maintain a good working state.

### Impact of role stress and psychological empowerment on organizational silence among psychiatric nurses: a person-centered approach

Building on the variable-centered approach, this study further adopted a person-centered perspective. The latent profile analysis results showed that the role stress–psychological empowerment patterns among psychiatric nurses could be divided into three latent classes: low stress–high empowerment (15.09%), moderate stress–moderate empowerment (62.66%), and high stress–low empowerment (22.25%). Analysis of variance and *post-hoc* comparisons indicated significant differences across these classes in total organizational silence and all its dimensions, thus supporting Hypothesis 3. First, the moderate stress–moderate empowerment class comprised the largest number of nurses (n = 245), suggesting that more than half of the psychiatric nurses in the hospital experienced moderate levels of both role stress and psychological empowerment, facing a certain degree of dual challenges. Therefore, attention should be paid to the development of this group, and appropriate guidance and support should be provided to prevent further transition toward the high stress–low empowerment class. Second, although the low stress–high empowerment class had a relatively small number of nurses (n = 59), they exhibited a lower tendency toward organizational silence. Their organizational silence scores were significantly lower, reflecting that psychological empowerment may play an important protective role in alleviating role stress and organizational silence among psychiatric nurses, which is consistent with previous findings (25, 26). In addition, this group can serve as a reference for an ideal state, providing direction for designing intervention strategies targeting other types of nurses. Finally, the analysis of variance and *post-hoc* tests revealed that nurses in the high stress–low empowerment class had significantly higher organizational silence scores than those in the other classes, indicating that this group was more likely to adopt organizational silence as a coping strategy. For this group, it is recommended that hospital managers pay close attention and implement effective interventions, such as adjusting job responsibilities, appropriately reducing workload, enhancing nurses’ sense of participation in organizational decision-making, and conducting targeted psychological stress reduction and empowerment activities, so as to reduce their tendency toward organizational silence and improve overall organizational health.

### Practical implications and future research

The practical implications of this study are reflected in two main levels. At the organizational level, managers can build a dual support environment of “stress reduction and empowerment”. On one hand, stress reduction should be addressed at the source by formulating clear job responsibilities and work procedures, which, according to role theory, is considered a structural approach to alleviating role stress (27). On the other hand, systematic empowerment should be implemented, for example, by promoting shared governance models to enhance nurses’ participation in clinical decision-making and department management, thereby increasing their work autonomy (51). Encouraging senior nurses to form teams with newly recruited nurses, through experience sharing and emotional support, not only facilitates skill development but also strengthens both parties’ recognition of the value of the nursing profession, thereby enhancing the sense of professional meaning (52) and improving the overall level of psychological empowerment. At the individual level, the classification results from LPA can be used to implement differentiated interventions. For nurses in the “high stress–low empowerment” class, focused attention and resource allocation should be provided, with interventions such as job redesign and one-on-one empowerment coaching. For the majority of nurses in the “moderate stress–moderate empowerment” class, regular group sharing sessions and peer support activities should be organized to prevent their transition toward unfavorable classes. Meanwhile, nurses in the “low stress–high empowerment” class should be valued and encouraged to serve as role models, thereby promoting a positive overall climate.

### Limitations

This study has several limitations that warrant further investigation. First, the cross-sectional design cannot determine causal relationships among the variables. Future research could adopt a longitudinal design to dynamically reveal the causal pathways between variables. Second, the sample was relatively limited, as the survey was conducted in only one tertiary psychiatric hospital. Future studies should expand the sample to include psychiatric hospitals in different regions, as well as across different cultures and countries, to explore possible differences and commonalities and thereby enhance the generalizability of the findings. Finally, because role stress had a significant direct effect on organizational silence and the mediation was only partial, this indirectly suggests that there may be other variables that play a role in the relationship between role stress and organizational silence behavior.

## Conclusions

The results of this study indicate that psychological empowerment plays a partial mediating role between role stress and organizational silence behavior among psychiatric nurses. Role stress not only directly affects organizational silence behavior but also exerts an indirect effect through psychological empowerment. Furthermore, nursing managers should not only pay attention to the influence of psychological empowerment but also focus on the synergistic effect of role stress and psychological empowerment. Targeted interventions should be implemented for different classes of nurses within the group to reduce and prevent the occurrence of organizational silence behavior.

## Data Availability

The original contributions presented in the study are included in the article/supplementary material. Further inquiries can be directed to the corresponding authors.

## References

[1] Kim MY Kim Y . Effectiveness of a patient safety incident disclosure education program: A quasi-experimental study. J Nurs Res. (2024) 32:e332. doi: 10.1097/jnr.0000000000000614 38814997

[2] Gan YD Di JZ Luo L Yu K Xu JM . Improving employee silence behavior through smart management. Chin Health Qual Manage. (2022) 29:96–9. doi: 10.13912/j.cnki.chqm.2022.29.03.25

[3] Park S Kim K Kim S Jones CB . Influence of workplace bullying and bystander types on speaking up for patient safety among hospital nurses: A cross-sectional study. J Adv Nurs. (2025) 81:6441–51. doi: 10.1111/jan.16788 39888086 PMC12460945

[4] Zhang WX Zhu HB Li J Zhang XM Wang S Yu J . Research progress on organizational silence among clinical nurses. Chin Nurs Manage. (2019) 19:579–83.

[5] Li ZY Yang YP Wang Q Tung TH Chen HX . The relationship between negative leadership behaviours and silence among nurses. J Nurs Manag. (2024) 2024:4561005. doi: 10.1155/2024/4561005 40224853 PMC11918819

[6] Zhang W Zhu Q Yu R He T Huang J . Regulatory focus as a mediator in the relationship between nurses' organizational silence and professional identity. J Adv Nurs. (2024) 80:3625–36. doi: 10.1111/jan.16113 38380591

[7] Lv X Gu Y Solomon OM Shen Y Ren Y Wei Y . Status and influencing factors of nurses' organizational silence in general hospitals in eastern coastal cities of China. BMC Nurs. (2024) 23:757. doi: 10.1186/s12912-024-02419-5 39407262 PMC11479549

[8] Zhu W Liao H Wu Q . Organizational silence and work engagement among Chinese clinical nurses: The mediating role of career calling. J Nurs Manag. (2025) 2025:7522633. doi: 10.1155/jonm/7522633 40547051 PMC12181662

[9] Abrams R Conolly A Rowland E Harris R Kent B Kelly D . Speaking up during the COVID-19 pandemic: Nurses' experiences of organizational disregard and silence. J Adv Nurs. (2023) 79:2189–99. doi: 10.1111/jan.15526 36645162

[10] Zou J Zhu X Fu X Zong X Tang J Chi C . The experiences of organizational silence among nurses: A qualitative meta-synthesis. BMC Nurs. (2025) 24:31. doi: 10.1186/s12912-024-02636-y 39789532 PMC11715701

[11] Jang SJ Son YJ Lee H . Prevalence, associated factors and adverse outcomes of workplace violence towards nurses in psychiatric settings: A systematic review. Int J Ment Health Nurs. (2022) 31:450–68. doi: 10.1111/inm.12951 34773361

[12] Yang XQ . The Relationship Between Workplace Violence and Silence Behavior Among Medical Staff: The Mediating Role of Emotional Labor Strategies. Harbin, China: Harbin Engineering University (2017).

[13] Maddineshat M Khodaveisi M Kamyari N Razavi M Pourmoradi F Sadeghian E . Exploring the safe environment provided by nurses in inpatient psychiatric wards: A mixed-methods study. J Psychiatr Ment Health Nurs. (2024) 31:257–69. doi: 10.1111/jpm.12983 37740710

[14] Kritsotakis G Gkorezis P Andreadaki E Theodoropoulou M Grigoriou G Alvizou A . Nursing practice environment and employee silence about patient safety: The mediating role of professional discrimination experienced by nurses. J Adv Nurs. (2022) 78:434–45. doi: 10.1111/jan.14994 34337760

[15] He L Liu J Sun R Deng Y Tang L Chen S . Mediating effect of emotional labour on the role pressure and silence behaviour of nurses. Eval Health Prof. (2026) 49(1):3–9. doi: 10.1177/01632787251329029 40170229

[16] Wu M Peng Z Estay C . How role stress mediates the relationship between destructive leadership and employee silence: The moderating role of job complexity. J Pac Rim Psychol. (2018). doi: 10.1017/prp.2018.741292463

[17] Roy DF Kahn RL Wolfe DM Quinn RP Snoek JD Rosenthal RS . Organizational Stress : Studies in Role Conflict and Ambiguity. (1964).

[18] Huang X Wang L Dong X Li B Wan Q . Effects of nursing work environment on work-related outcomes among psychiatric nurses: A mediating model. J Psychiatr Ment Health Nurs. (2021) 28:186–96. doi: 10.1111/jpm.12665 32557979

[19] Li HQ Xie P Jiang W Hou Y Gong S Jin S . Psychiatric nurses' preferences for job: a discrete choice experiment. BMC Nurs. (2025) 24:240. doi: 10.1186/s12912-025-02862-y 40033292 PMC11877894

[20] Zhang X Zhang L Xue B Li Y Yan M Luo H . Effort-reward imbalance and well-being among psychiatric nurses: The mediating role of burnout and decent work. BMC Nurs. (2024) 23:635. doi: 10.1186/s12912-024-02301-4 39256745 PMC11389592

[21] Mensah DK . Perspectives of psychiatric nurses on the stigmatization of mental healthcare in Ghana: a qualitative study. Front Public Health. (2024) 12:1423445. doi: 10.3389/fpubh.2024.1423445 39525470 PMC11543473

[22] Biddle BJ . Recent developments in role theory. Annu Rev Sociol. (1986) 12:67–92. doi: 10.1146/annurev.so.12.080186.00043541139587

[23] Anglin AH Kincaid PA Short JC Allen DG . Role theory perspectives: Past, present, and future applications of role theories in management research. J Manage. (2022). doi: 10.1177/01492063221081442

[24] Spreitzer GM . Psychological empowerment in the workplace: Dimensions, measurement, and validation. Acad Manage J. (1995) 38:1442–65. doi: 10.5465/256865

[25] Jia J Chen WZ . The impact of psychological empowerment on employee role stress. J Southwest Pet Univ (Soc Sci Ed). (2011) 13:69–74.42491485

[26] Chatziioannou A Bochoridou A Gkorezis P Kantouni A . Breaking the silence in the health-care sector: the relationship between empowering leadership and nurses' silence toward patient safety via psychological empowerment. Leadersh Health Serv. (2025) 38:339–55. doi: 10.1108/LHS-07-2024-0069 40052740

[27] Demerouti E Bakker AB Nachreiner F Schaufeli WB . The job demands-resources model of burnout. J Appl Psychol. (2001) 86:499–512. doi: 10.1037/0021-9010.86.3.499 11419809

[28] Bakker AB Demerouti E . Job demands-resources theory: Taking stock and looking forward. J Occup Health Psychol. (2017) 22:273–85. doi: 10.1037/ocp0000056 27732008

[29] Mori M Krumholz HM Allore HG . Using latent class analysis to identify hidden clinical phenotypes. JAMA. (2020) 324:700–1. doi: 10.1001/jama.2020.2278 32808993

[30] Qiu HZ . Principles and Techniques of Latent Class Models. Beijing: Education Science Press (2008).

[31] Wen ZL Xie JY Wang HH . Principles, procedures, and implementation of latent class models. J East China Norm Univ (Educ Sci Ed). (2023) 41:1–15. doi: 10.16382/j.cnki.1000-5560.2023.01.001

[32] Ni P Chen JL Liu N . Sample size estimation in quantitative nursing research. Chin J Nurs. (2010) 45:378–80.

[33] Schumacker RE Lomax RG . A Beginner’s Guide to Structural Equation Modeling. Mahwah: Lawrence Erlbaum Associates (1996).

[34] Nylund-Gibson K Garber AC Carter DB Chan M Arch DAN Simon O . Ten frequently asked questions about latent transition analysis. Psychol Methods. (2023) 28:284–300. doi: 10.1037/met0000486 35834194

[35] Mukaihata T Kato Y Swa T Fujimoto H . Work engagement of psychiatric nurses: a scoping review. BMJ Open. (2024) 14:e081941. doi: 10.1136/bmjopen-2023-081941 38684255 PMC11086197

[36] Lu Q Wang M Zuo Y Tang Y Zhang R Zhang J . Construction and verification of a risk prediction model of psychological distress in psychiatric nurses. BMC Nurs. (2025) 24:161. doi: 10.1186/s12912-025-02796-5 39934812 PMC11817827

[37] Li CP Zhang Y . The impact of role stressors on teachers' physical health and mental health. Adv Psychol Sci. (2009) 25:114–9. doi: 10.16187/j.cnki.issn1001-4918.2009.01.017

[38] Dziedzic B Łodziana K Marcysiak M Kryczka T . Research on the relationship between clinical nurses' role stress and social support. J Nurs Manag. (2016) 16:627–9.

[39] Orgambídez A Almeida H . Exploring the link between structural empowerment and job satisfaction through the mediating effect of role stress: A cross-sectional questionnaire study. Int J Nurs Stud. (2020) 109:103672. doi: 10.1016/j.ijnurstu.2020.103672 32569828

[40] Li CP Li XX Shi K Chen XF . The measurement of empowerment and its relationship with employee work attitudes. Acta Psychol Sin. (2006) 38:99–106.

[41] Shi XM Liu ML Fan H Li YP . A study on the correlation between psychological empowerment, professional identity and job burnout of psychiatric nurses. Anhui Med J. (2024) 45:492–6. doi: 10.3969/j.issn.1000-0399.2024.04.019

[42] Yang J . Preliminary Development of a Nurse Organizational Silence Assessment Questionnaire. Taiyuan: Shanxi Medical University (2016).

[43] Yang J Yang H . Developing a nurse organizational silence assessment questionnaire using the Delphi method. Chin Nurs Res. (2017) 31:2356–8.

[44] Zhou H Long LR . Statistical remedies for common method biases. Adv Psychol Sci. (2004) 12:942–50. doi: 10.3969/j.issn.1671-3710.2004.06.018

[45] Wang MC Deng QW Bi XY Ye HS Yang WD . The performance of the entropy-based classification accuracy index in latent profile analysis: A Monte Carlo simulation study. Acta Psychol Sin. (2017) 49:1473–82. doi: 10.3724/SP.J.1041.2017.01473

[46] Weller BE Bowen NK Faubert SJ . Latent class analysis: a guide to best practice. J Black Psychol. (2020) 46:287–311. doi: 10.1177/009579842093093237652644

[47] Lambert VA Lambert CE . Literature review of role stress/strain on nurses: an international perspective. Nurs Health Sci. (2001) 3:161–72. doi: 10.1046/j.1442-2018.2001.00086.x 11882192

[48] Kong Y Zheng YH Liu Y Lu JT Zhang JS Chen RB . The chain mediating effects of professional identity and coping style between personal mastery and psychological empowerment among clinical nurses. J Nurs Manag. (2025) 25:397–403. doi: 10.3969/j.issn.1671-315x.2025.05.007

[49] Fan H Shen YH Wu XM Ming L Liu M . The influence of psychological empowerment of mental health nurses on job burnout and the mediating role of anxiety level. Occup Health. (2022) 38:2319–23. doi: 10.13329/j.cnki.zyyjk.2022.0485

[50] Tomura M . Psychiatric nurses' experience of moral distress: its relationship with empowerment and coping. Nurs Ethics. (2023) 30:1095–113. doi: 10.1177/09697330231153915 37226471

[51] Yin Y Feng J Lu F Guo N Wang H . Theory-guided nursing management practices at Stanford Health Care in the United States and their implications. Mil Nurs. (2025) 42:109–12. doi: 10.3969/j.issn.2097-1826.2025.09.026

[52] Xu Y Liu WJ Wang X Yang QM . Occupational protection behavior and its influencing factors of newly recruited nurses. BMC Med Educ. (2023) 23:797. doi: 10.1186/s12909-023-04780-6 37880657 PMC10601324

